# Increasing prevalence of hypertension among HIV-positive and negative adults in Senegal, West Africa, 1994-2015

**DOI:** 10.1371/journal.pone.0208635

**Published:** 2018-12-31

**Authors:** Noelle A. Benzekri, Moussa Seydi, Ibrahima N. Doye, Macoumba Toure, Marie Pierre Sy, Nancy B. Kiviat, Papa Salif Sow, Geoffrey S. Gottlieb, Stephen E. Hawes

**Affiliations:** 1 Department of Medicine, University of Washington, Seattle, WA, United States of America; 2 Services des Maladies Infectieuses et Tropicales, Centre Hospitalier Universitaire de Fann, Dakar, Senegal; 3 Conseil National de Lutte contre le Sida, Dakar, Senegal; 4 Department of Pathology, University of Washington, Seattle, WA, United States of America; 5 Department of Global Health, University of Washington, Seattle, WA, United States of America; 6 Department of Epidemiology, University of Washington, Seattle, WA, United States of America; The University of Warwick, UNITED KINGDOM

## Abstract

**Background:**

Non-communicable diseases, including hypertension (HTN), are increasingly recognized as important causes of morbidity and mortality among people living with HIV (PLHIV) in resource-limited settings. The goals of this study were to determine the prevalence of HTN among PLHIV in Senegal over time and to identify predictors of HTN among HIV-positive versus HIV-negative adults.

**Methods:**

We conducted a retrospective study using data from individuals enrolled in previous studies in Senegal from 1994–2015. Blood pressure (BP) measurements taken during study visits were used for analysis. HTN was defined as systolic BP≥140 or diastolic BP≥90. We used logistic regression to identify predictors of HTN.

**Results:**

We analyzed data from 2848 adults (1687 HIV-positive, 1161 HIV-negative). Among PLHIV, the prevalence of HTN increased from 11% during 1994–1999 to 22% during 2010–2015. Among HIV-negative individuals, the prevalence of HTN increased from 16% to 32%. Among both groups, the odds of HTN more than doubled from 1994–1999 to 2010–2015 (HIV-positive OR 2·4, 95% CI 1·1–5·0; HIV-negative OR 2·6, 95% CI 1·5–4·6). One quarter of all individuals with HTN had stage 2 HTN. The strongest risk factor for HTN was obesity (HIV-positive OR 3·2, 95% CI 1·7–5·8; p<0·01; HIV-negative OR 7·8, 95% CI 4·5–13·6; p<0·01). Male sex and age ≥50 were also predictive of HTN among both groups. Among HIV-positive subjects, WHO stage 1 or 2 disease was predictive of HTN and among HIV-negative subjects, having no formal education was predictive.

**Conclusion:**

Over the past 20 years, the prevalence of HTN has doubled among both HIV-positive and HIV-negative adults in Senegal. Our study indicates that there is an increasing need for the integration of chronic disease management into HIV programs in Senegal. Furthermore, our findings highlight the need for enhanced prevention, recognition, and management of non-communicable diseases, including hypertension and obesity, among both HIV-positive and HIV-negative individuals in Senegal.

## Introduction

Global antiretroviral therapy (ART) coverage has reached 46%, resulting in a 26% decline in AIDS-related deaths globally since 2010 [1]. With the increasing availability of ART, people living with HIV (PLHIV) are living longer and are increasingly confronted by the burden of non-communicable diseases (NCD), including hypertension (HTN) [1–5]. Hypertension is a leading risk factor for mortality and disability worldwide [6–11]. Although data regarding the changes in the epidemiology of HTN in sub-Saharan Africa are limited relative to other regions of the world, recent studies have demonstrated that both mean blood pressure and the prevalence of hypertension are increasing in the region [12–16]. Recent estimates suggest that many countries in West Africa, including Senegal, are now among the highest ranked globally in terms of prevalence of HTN in the general population [12].

Although nearly 70% of the world’s HIV-positive population lives in sub-Saharan Africa [1] and more than 80% of global premature deaths due to NCD occur in low and middle-income countries [6], the majority of studies evaluating the epidemiology of HTN among PLHIV have been conducted in high-income countries [17–21]. Data regarding the epidemiology of HTN among PLHIV in sub-Saharan Africa are limited, and there have been no studies evaluating changes in the prevalence of HTN among PLHIV in sub-Saharan Africa over time. Such studies are needed to guide national programs, develop effective interventions, and set priorities for resource allocation.

We used over 20 years of data to evaluate changes in the prevalence of HTN in Senegal, West Africa and to identify risk factors for HTN among HIV-positive versus HIV-negative individuals.

## Methods

We conducted a retrospective study using data from individuals enrolled in observational studies in outpatient clinics in Senegal from 1994–2015 (see **S1 Supporting Information** for a description of parent studies) [22–26]. Participants in all parent studies provided written informed consent. Study procedures for all parent studies were approved by the University of Washington Institutional Review Board and the Senegal Comité National d'Ethique pour la Recherche en Santé. Data from each individual’s initial encounter were included. Pre-hypertension was defined as systolic blood pressure (SBP) 120–139 or diastolic blood pressure (DBP) 80–89. Hypertension was defined as SBP ≥140 or DBP ≥90. Stage 2 hypertension was defined as SBP ≥160 or DBP ≥100. Nutritional status was determined by BMI (kg/m^2^), whereby a BMI <18.5 was classified as underweight, a BMI of 18.5–24.9 was considered normal weight, overweight was defined as a BMI of 25.0–29.9, and a BMI ≥30.0 was classified as obese.

Data were analyzed using SPSS Statistics 23 (IBM, Armonk, N.Y.). Descriptive analysis was performed for all variables. Chi-square tests were used to identify differences in categorical variables between groups. The Mann-Whitney U test was used to identify differences in medians between groups. Logistic regression was used to identify predictors of hypertension among HIV-negative and HIV-positive individuals. Variables identified in the literature as predictive of hypertension were evaluated and included in the multiple regression model, in addition to variables that were identified as predictive using simple regression. For the categorical variable “year”, the reference category was 1994–1999. For the categorical variable “age”, the reference category was age 18–34. For the variable “BMI category”, the reference was normal BMI. Missing data were excluded from analysis. P-values <0.05 were considered significant.

## Results

A total of 2848 individuals were included in this analysis, of which 1687 were HIV-positive and 1161 were HIV-negative **(Table 1)**. The majority of subjects (75.4%) were female. Age ranged from 18–84 years, with a median of 37 years among HIV-positive subjects and a median of 33 among HIV-negative subjects.

**Table 1:** Participant characteristics and comparison according to HIV status.

	Combined n (%)	HIV positive n (%)	HIV negative n (%)	p- value
**N, number of individuals**	2848	1687 (59.2)	1161 (40.8)	
**Female**	2141 (75.4)	**1172 (69.6)**	**969 (83.8)**	**<0.01**
**Age, median years (range)**	35 (18–84)	**37 (18–72)**	**33 (18–84)**	**<0.01**
**Age categories**				**<0.01**
18–34	1302 (46.2)	**677 (40.5)**	**625 (54.4)**	**-**
35–49	1201 (42.6)	**798 (47.7)**	**403 (35.1)**	**-**
50+	318 (11.3)	**197 (11.8)**	**121 (10.5)**	**-**
**Marital status**				**<0.01**
Single	518 (18.5)	**261 (15.8)**	**257 (22.5)**	**-**
Monogamous	869 (31.1)	**506 (30.6)**	**363 (31.8)**	**-**
Polygamous	492 (17.6)	**234 (14.2)**	**258 (22.6)**	**-**
Divorced	677 (24.2)	**454 (27.5)**	**223 (19.5)**	**-**
Widowed	240 (8.6)	**198 (12.0)**	**42 (3.7)**	**-**
Children*				**<0.01**
0–2	872 (41.3)	**491 (42.7)**	**381 (39.6)**	**-**
3–5	738 (34.9)	**428 (37.2)**	**310 (32.2)**	**-**
6+	503 (23.8)	**232 (20.2)**	**271 (28.2)**	**-**
Use contraception*^,^^$^	765 (39.0)	**313 (31.2)**	**452 (47.2)**	**<0.01**
Use OCP	185 (9.4)	**67 (6.7)**	**118 (12.3)**	**<0.01**
**No education**	1320 (47.5)	**873 (53.2)**	**447 (39.3)**	**<0.01**
**Smoking**^$^	626 (22.5)	**411 (25.0)**	**215 (18.8)**	**<0.01**
**Alcohol use**^$^	337 (12.1)	**240 (14.6)**	**97 (8.5)**	**<0.01**
**Commercial sex worker**	728 (25.6)	**460 (27.3)**	**268 (23.1)**	**0.01**
**BMI, median (range)**	20.5 (10.2–44.5)	**19.5 (10.2–42.9)**	**22.0 (11.4–44.5)**	**<0.01**
**BMI category**				**<0.01**
Underweight	739 (30.8)	**572 (39.7)**	**167 (17.4)**	**-**
Overweight	345 (14.4)	**161 (11.2)**	**184 (19.2)**	**-**
Obese	177 (7.4)	**77 (5.3)**	**100 (10.4)**	**-**
**Pre-hypertension**^**a**^	1681 (59.1)	**968 (57.4)**	**713 (61.7)**	**0.02**
**Hypertension**^**b**^	405 (14.2)	**201 (11.9)**	**204 (17.6)**	**<0.01**
Stage 2 hypertension^c^	100 (25.0)^d^	50 (24.9)^d^	50 (25.1)^d^	0.95
**SBP, median (range)**	120 (80–220)	**120 (80–200)**	**120 (90–220)**	**<0.01**
**DBP, median (range)**	80 (40–130)	80 (40–130)	80 (40–120)	0.06
**HIV-associated factors**				
WHO stage 3 or 4		684 (43.5)		
CD4, median (range)		281 (1–2000)		
CD4<200/mm^3^		534 (40.0)		
On ART		161 (11.0)		

^a^Pre-hypertension: Systolic blood pressure (SBP) 120–139 or diastolic blood pressure (DBP) 80–89

^b^Hypertension: SBP ≥140 or DBP ≥90

^c^Stage 2 hypertension: SBP ≥160 or DBP ≥100. ^d^Percent of hypertensive subjects with stage 2 hypertension.

*Data available for female subjects only.

^$^When excluding CSW: 320 (23.6%) of all subjects, 88 (13.3%) of HIV-positive subjects and 232 (33.5%) of HIV-negative subjects used contraception (p<0.01); 170 (14.3%) of HIV-positive subjects and 87 (9.8%) of HIV-negative subjects were smokers (p<0.01); 75 (6.3%) of HIV-positive subjects and 26 (2.9%) of HIV-negative subjects used alcohol (p<0.01).

Among HIV-positive subjects, 15.8% were single, 30.6% were monogamous, 14.2% were polygamous, 27.5% were divorced, and 12.0% were widowed. Among HIV-negative subjects, 22.5% were single, 31.8% were monogamous, 22.6% were polygamous, 19.5% were divorced, and 3.7% were widowed. Nearly a quarter of female subjects had 6 or more children and the majority (61.0%) did not use contraception. Almost half of all subjects had no formal education. A quarter of HIV-positive subjects were smokers versus 18.8% of HIV-negative subjects, and approximately 14.6% of HIV-positive subjects used alcohol versus 8.5% of HIV-negative subjects. Approximately a quarter of female subjects were commercial sex workers. When excluding commercial sex workers, 23.6% of all subjects, 13.3% of HIV-positive subjects and 33.5% of HIV-negative subjects used contraception; 14.3% of HIV-positive subjects and 9.8% of HIV-negative subjects were smokers, and 6.3% of HIV-positive subjects and 2.9% of HIV-negative subjects used alcohol.

The median BMI among HIV-positive subjects was 19.5 versus 22.0 among HIV-negative subjects. Approximately 16.5% of HIV-positive subjects were overweight or obese versus 30% of HIV-negative subjects. Among HIV-positive subjects nearly 40% were underweight versus 17.4% of HIV-negative subjects.

Approximately 60% of all subjects had pre-hypertension. Among HIV-positive individuals, 11.9% had hypertension, of which 8.5% had systolic HTN and 9.0% had diastolic hypertension. Among HIV-negative individuals, 17.6% had hypertension, of which 13.3% had systolic HTN and 11.2% had diastolic hypertension. One quarter of both HIV-positive and HIV-negative individuals with HTN had stage 2 HTN. Among HIV-positive individuals, systolic blood pressure (SBP) ranged from 80–200 and diastolic blood pressure (DBP) ranged from 40–130, versus a SBP range of 90–220 and a DBP range of 40–120 among HIV-negative individuals.

Among HIV-positive subjects, 43.5% had WHO stage 3 or 4 disease, 40.0% had a CD4 count <200/mm^3^, and 11.0% were on antiretroviral therapy (ART). The odds of receiving ART increased with time (OR 1.89 per year, 95% CI 1.71–2.10; p<0.01).

**Fig 1:**
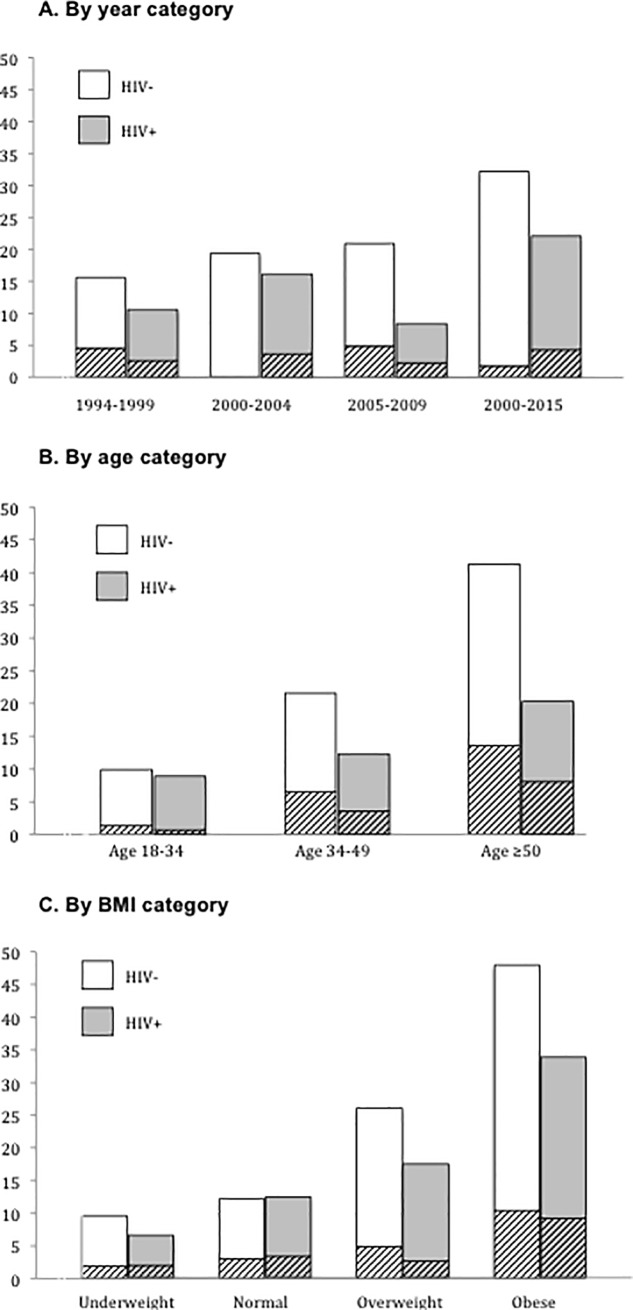
Prevalence (%) of hypertension among HIV-positive and HIV-negative subjects. **Fig 1A.** X-axis: year category; **Fig 1B.** X-axis: age category; **Fig 1C.** X-axis: BMI category. For all figures, Y-axis = prevalence of HTN (SBP ≥140 or DBP ≥90) and striped portions indicate subset with stage 2 HTN (SBP ≥160 or DBP ≥100).

Among HIV-positive individuals, the overall prevalence of hypertension increased from 10.7% in 1994–1999, to 22.2% in 2010–2015 (p<0.01) **(Fig 1A)**. Among HIV-negative individuals, the overall prevalence of hypertension increased from 15.6% in 1994–1999, to 32.2% in 2010–2015 (p<0.01). Among both HIV-positive and HIV-negative subjects the prevalence of hypertension increased with age **(Fig 1B)**. Among HIV-positive individuals the prevalence of HTN was 8.9% for those age 18–34, 12.3% for those 35–49, and 20.3% for those ≥50 (p<0.01). For HIV-negative individuals, the prevalence of HTN was 9.9% among those age 18–34, 21.6% for those 35–49, and 41.3% for those ≥50 (p<0.01). The prevalence of hypertension increased with BMI for both HIV-positive and HIV-negative subjects **(Fig 1C)**. Among HIV-positive individuals, the prevalence of HTN was 6.5% for underweight subjects, 12.4% for normal weight subjects, 17.4% for overweight subjects, and 33.8% among obese subjects (p<0.01). Among HIV-negative individuals, the prevalence of hypertension was 9.6% for underweight subjects, 12.2% for normal weight subjects, 26.1% among overweight subjects, and 48.0% among obese subjects (p<0.01).

HIV-negative individuals had greater odds of hypertension compared to HIV-positive individuals (OR 1.6, 95% CI 1.3–2.0; p<0.01) **(Table 2)**. Among HIV-positive individuals, WHO stage 1 or 2 disease and CD4 cell count ≥200 were predictive of hypertension. ART status was not predictive of hypertension.

**Table 2:** Simple logistic regressions evaluating HIV-status, WHO stage, CD4 count, and ART as predictors of hypertension.

Simple regressions				
	OR	95% CI		p-value
**HIV status negative**	**1.58**	1.28	1.95	**<0.01**
WHO stage 1 or 2*	**2.09**	1.50	2.91	**<0.01**
CD4 count ≥200*	**1.95**	1.36	2.78	**<0.01**
On ART*	0.72	0.41	1.28	0.265

*Among HIV-positive subjects only

Among HIV-positive subjects, year, age, and BMI, were predictive of hypertension in simple regression analysis and the odds of HTN more than doubled from the time period of 1994–1999 to the time period of 2010–2015 **(Table 3)**. Data on number of children was only available for female subjects. Women with 6 or more children had greater odds of hypertension. This relationship persisted when controlling for age.

**Table 3:** Simple regressions evaluating predictors of hypertension among A. HIV-positive and B. HIV-negative participants.

Simple regressions								
	A. HIV-positive				B. HIV-negative			
	OR	95% CI		p-value	OR	95% CI		p-value
**Year category**^**a**^								
2000–2004	**1.62**	1.15	2.27	**<0.01**	1.30	0.52	3.24	0.57
2005–2009	0.77	0.50	1.17	0.22	1.43	0.99	2.06	0.06
2010–2015	**2.38**	1.14	4.96	**0.02**	**2.58**	1.45	4.60	**<0.01**
**Male**	0.95	0.69	1.32	0.77	1.11	0.74	1.66	0.62
**Age category**^**b**^								
35–49	**1.44**	1.03	2.02	**0.04**	**2.50**	1.76	3.56	**<0.01**
50+	**2.62**	1.69	4.06	**<0.01**	**6.40**	4.09	10.00	**<0.01**
**BMI category**^**c**^								
Underweight	0.49	0.33	0.74	<0.01	0.76	0.43	1.36	0.36
Overweight	1.49	0.93	2.39	0.10	**2.53**	1.66	3.87	**<0.01**
Obese	**3.61**	2.13	6.13	**<0.01**	**6.63**	4.13	10.64	**<0.01**
**Number of children**^**d**^^,^^**e**^								
3–5	1.28	0.83	1.98	0.26	**2.18**	1.37	3.46	**<0.01**
6+	**2.65**	1.70	4.13	**2.65**	**4.42**	2.84	6.87	**<0.01**
**No education**	0.94	0.70	1.27	0.94	**1.77**	1.29	2.41	**<0.01**
**Smoking**	0.73	0.51	1.06	0.73	0.48	0.30	0.77	<0.01
**Alcohol use**	1.31	0.886	1.949	1.31	1.16	0.686	1.966	0.58

^a^Reference: 1994–1999.

^b^Reference: age 18–34.

^c^Reference: normal BMI.

^d^Reference: 0–2 children; data on number of children available only for female subjects.

^e^Women with 6 or more children had greater odds of hypertension when controlling for age: HIV-negative subjects: OR 1.77, 95% CI 1.03–3.06; p = 0.04; HIV-positive subjects: OR 2.18, 95% CI 1.33–3.55; p<0.01.

In multiple regression analysis, among HIV-positive individuals, male sex (OR 2.19, 95% CI 1.37–3.51; p<0.01), age ≥50 (OR 2.26, 95% CI 1.30–3.93; p<0.01), obesity (OR 3.17, 95% CI 1.74–5.76; p<0.01), and WHO stage 1 or 2 (OR 1.99, 95% CI 1.24–3.18; p<0.01) were predictive of hypertension **(Table 4).** Among HIV-negative subjects, male sex (OR 2.21, 95% CI 1.30–3.76; p<0.01), age 35–49 (OR 1.88, 95% CI 1.22–2.90; p<0.01), age ≥50 (OR 4.89, 95% CI 2.61–9.16; p<0.01), overweight (OR 3.07, 95% CI 1.89–4.96; p<0.01), obesity (OR 7.81, 95% CI 4.49–13.56; p<0.01), and having no formal education (OR 1.61, 95% CI 1.10–2.34; p = 0.01), were predictive of hypertension.

**Table 4:** Multiple regressions evaluating predictors of hypertension among A. HIV- positive and B. HIV-negative participants.

Multiple regressions*								
	A. HIV-positive (N = 1364)				B. HIV-negative (N = 935)			
	OR	95% CI		p-value	OR	95% CI		p-value
**Male**	**2.19**	1.37	3.51	**<0.01**	**2.21**	1.30	3.76	**<0.01**
**Age category**								
35–49	1.33	0.88	2.00	0.18	**1.88**	1.22	2.90	**<0.01**
50+	**2.26**	1.30	3.93	**<0.01**	**4.89**	2.61	9.16	**<0.01**
**BMI category**								
Underweight	0.57	0.36	0.90	**0.02**	0.58	0.31	1.11	0.10
Overweight	1.47	0.89	2.42	0.13	**3.07**	1.89	4.96	**<0.01**
Obese	**3.17**	1.74	5.76	**<0.01**	**7.81**	4.49	13.56	**<0.01**
**No education**	**-**	-	-	-	**1.61**	1.10	2.34	**0.01**
**WHO stage 1 or 2** (reference WHO stage 3 or 4)	**1.99**	1.24	3.18	**<0.01**	**-**	-	-	**-**

*Controlling for year

## Discussion

We found that over the past twenty years, the prevalence of hypertension has doubled among both HIV-positive and HIV-negative individuals in Senegal. Importantly, among those who were hypertensive, a quarter had stage 2 hypertension, suggesting inadequate disease recognition and management. Furthermore, the majority of both HIV-positive and HIV-negative participants had pre-hypertension. Our findings contribute to the growing body of literature indicating that NCD represent an emerging public health challenge in sub-Saharan Africa [12–16].

Previous studies have demonstrated that the prevalence of hypertension in the general population is increasing in sub-Saharan Africa [12–15] and the region of West Africa is at especially high risk [11–14]. For more than half the countries in the West Africa, the prevalence of HTN is >30% [12]. This is consistent with our finding that by 2010–2015, the overall prevalence of HTN among HIV-negative subjects was 32%.

The prevalence of HTN among HIV-positive subjects in our study increased from approximately 11% in 1994–1999, to more than 22% in 2010–2015. Although data regarding the epidemiology of hypertension over time among PLHIV in sub-Saharan Africa are limited and represent heterogeneous populations [27–50], the majority of previous studies report a prevalence of HTN among PLHIV ranging from 20–35%. Similar to previous studies conducted in sub-Saharan Africa [27–52], we found that male sex, age, and BMI were risk factors for HTN among both HIV-negative and HIV-positive subjects. Obese individuals and those ≥50 years of age were at especially high risk. By 2010–2015, more than half of HIV-negative subjects ≥50 years of age and nearly half of HIV-positive subjects ≥50 years of age, had elevated blood pressure.

Obesity was the strongest risk factor for hypertension and was associated with a threefold to eightfold increase in the odds of hypertension compared to normal weight. With the rapidly increasing burden of overweight and obesity in sub-Saharan Africa [53, 54], including Senegal [55], urgent preventive measures, including enhanced dietary education and the promotion of physical activity, should be emphasized as critical components of patient care.

Approximately a quarter of all women in this study had six or more children and among both HIV-positive and HIV-negative women, having six or more children was predictive of hypertension. Importantly, less than half of the women in this study used any form of contraception and among HIV-positive women, less than a third used contraception. Ensuring universal access to family planning services is recognized as a global health priority and human right by the international community [56–59]. Our findings are consistent with previous studies which report an unmet need for modern family planning in Senegal and West Africa [60, 61]. Addressing this gap should be a top priority for health programs in the region.

An important and modifiable risk factor for poor health outcomes was the lack of any formal education for nearly half of all participants. HIV-negative subjects who had not received any education were nearly twice as likely to be hypertensive than those who had received any form of formal education, including solely primary school. This finding is consistent with previous studies which have shown that lack of education is a risk factor for hypertension [34, 35, 52, 62–64]. The links between education and improvements in health are well-established [7, 65] and the promotion of the universal availability of education must be a priority for all national health programs.

Numerous studies have evaluated the association between ART and hypertension, though the results have been inconsistent [18, 20, 66]. In our study, we did not find an association between ART and hypertension. This finding is limited by the fact that only 11% of HIV-positive subjects were receiving ART. The low level of coverage among subjects in our study is not representative of current ART coverage in Senegal, with recent country estimates ranging from 31–56% [67, 68]. Relative to other regions of the world, gains in ART coverage have been slower in countries of Western and Central Africa, where regional coverage has reached only 28% [1].

As countries in West Africa, including Senegal, continue to make progress towards universal ART coverage and resultant gains in life expectancy are achieved, HIV programs must be prepared to address the growing burden of NCD among PLHIV. Furthermore, individual governments and international partners must continue to strive towards universal access to education and modern family planning.

Our study had several limitations. This was a retrospective study and the data were pooled from multiple prior research studies at urban clinics near Dakar, which in some instances oversampled specific sub-groups such as women, commercial sex workers and ART-naive HIV-infected individuals. Thus, our results may not represent associations which would be observed in individuals from the general population of Senegal. Given that we used data from a single study encounter, we were unable to evaluate variation in blood pressure over time, which may have resulted in under- or over-estimation of hypertension. This study would have been strengthened by the availability of data regarding treatment for hypertension and the presence of other comorbidities including other cardiovascular diseases, hyperlipidemia, and diabetes. This is an important limitation and calls for prospectively designed studies to better identify and quantify risk factors for non-communicable diseases among PLHIV in Sub-Saharan Africa.

## Conclusion

We utilized over 20 years of data to compare the epidemiology of hypertension among HIV-positive versus HIV-negative individuals in Senegal and provide the first evaluation of trends in hypertension over time among PLHIV in sub-Saharan Africa. We found that the burden of hypertension is increasing rapidly among both HIV-positive and HIV-negative individuals in Senegal. Obesity is a strong predictor of hypertension and is an important target for interventional strategies. Our findings demonstrate that NCD, including HTN, are increasingly important co-morbidities among PLHIV in sub-Saharan Africa and that the prevention, diagnosis, and management of NCD must be incorporated into HIV-programs.

## Supporting information

S1 Supporting Information Table A: Description of parent studies. Table B: Parent study aims and subjects.
(DOCX)
