# Correction: Increasing prevalence of hypertension among HIV-positive and negative adults in Senegal, West Africa, 1994-2015

**DOI:** 10.1371/journal.pone.0212250

**Published:** 2019-02-07

**Authors:** Noelle A. Benzekri, Moussa Seydi, Ibrahima N. Doye, Macoumba Toure, Marie Pierre Sy, Nancy B. Kiviat, Papa Salif Sow, Geoffrey S. Gottlieb, Stephen E. Hawes

There is an error in Table 3. The p-vales in column 5 for HIV-positive participants should be listed as follows: 6+ children, p-value <0.01; No education, p-value 0.68; Smoking, p-value 0.10; Alcohol use, p-value 0.18.

**Table 3:** Simple regressions evaluating predictors of hypertension among A. HIV-positive and B. HIV-negative participants.

Simple regressions								
	A. HIV-positive				B. HIV-negative			
	OR	95% CI		p-value	OR	95% CI		p-value
**Year category**^a^								
2000–2004	**1.62**	1.15	2.27	**<0.01**	1.30	0.52	3.24	0.57
2005–2009	0.77	0.50	1.17	0.22	1.43	0.99	2.06	0.06
2010–2015	**2.38**	1.14	4.96	**0.02**	**2.58**	1.45	4.60	**<0.01**
**Male**	0.95	0.69	1.32	0.77	1.11	0.74	1.66	0.62
**Age category**^b^								
35–49	**1.44**	1.03	2.02	**0.04**	**2.50**	1.76	3.56	**<0.01**
50+	**2.62**	1.69	4.06	**<0.01**	**6.40**	4.09	10.00	**<0.01**
**BMI category**^c^								
Underweight	0.49	0.33	0.74	<0.01	0.76	0.43	1.36	0.36
Overweight	1.49	0.93	2.39	0.10	**2.53**	1.66	3.87	**<0.01**
Obese	**3.61**	2.13	6.13	**<0.01**	**6.63**	4.13	10.64	**<0.01**
**Number of children**^d^^**,**^^e^								
3–5	1.28	0.83	1.98	0.26	**2.18**	1.37	3.46	**<0.01**
6+	**2.65**	1.70	4.13	**<0.01**	**4.42**	2.84	6.87	**<0.01**
**No education**	0.94	0.70	1.27	0.68	**1.77**	1.29	2.41	**<0.01**
**Smoking**	0.73	0.51	1.06	0.10	0.48	0.30	0.77	<0.01
**Alcohol use**	1.31	0.89	1.95	0.18	1.16	0.69	1.97	0.58

^a^Reference: 1994–1999.

^b^Reference: age 18–34.

^c^Reference: normal BMI.

^d^Reference: 0–2 children; data on number of children available only for female subjects.

^e^Women with 6 or more children had greater odds of hypertension when controlling for age: HIV-negative subjects: OR 1.77, 95% CI 1.03–3.06; p = 0.04; HIV-positive subjects: OR 2.18, 95% CI 1.33–3.55; p<0.01.
