# Supplementary material for: Increasing prevalence of hypertension among HIV-positive and negative adults in Senegal, West Africa, 1994-2015
Source: PLoS One. 2018 Dec 31;13(12):e0208635. doi: 10.1371/journal.pone.0208635 (PMC6312281; doi:10.1371/journal.pone.0208635)
Supplement: S1 Supporting Information — Table A: Description of parent studies. Table B: Parent study aims and subjects. (DOCX) [file pone.0208635.s001.docx]

**S1: Supporting Information**

**Table A in S1: Description of parent studies**

|  | **Study aims, description of subjects, and inclusion criteria** |
| --- | --- |
| **Parent study 1** | **Aims:** Compare the risk of development of CIN2-3 in relationship to HIV-1 and/or HIV-2 among HPV-positive women. Characterize the risk of development of CIN2-3 among HIV-infected women with respect to the role of HPV, specific types of HPV, and other STIs. Compare the occurrence of oral lesions in relation to HIV-1 and/or HIV-2 infection in men. |
|  | **Subjects:** Adult women and men presenting to an outpatient clinic in Dakar, and adult women at STI clinics for CSWs in Dakar and Mbour. |
| **Parent study 2** | **Aims:** Determine the association between HIV status and the practice of oral sex and other sexual practices. Describe the predictors of the presence and the quantity of HIV DNA and HIV RNA in oral secretions of HIV-1 and/or HIV-2 seropositive women. |
|  | **Subjects:** Adult female CSWs presenting to STI clinics in Dakar, Mbour and Sebikotane. |
| **Parent study 3** | **Aims:** Evaluate cellular immune responses in relation to HIV-1 and/or HIV-2 viral load and CD4 count. |
|  | **Subjects:** Adults presenting to outpatient clinics and STI clinics in Dakar. |
| **Parent study 4** | **Aims:** Identify a panel of hypermethylated genes that are predictive of CIN-3/ICC among both HIV-positive and HIV-negative women. Assess the risk of developing CIN-3 in relationship to HPV persistence, HIV, and the presence or acquisition of the candidate hypermethylated genes. |
|  | **Subjects:** Adult women presenting to outpatient clinics in Dakar and Pikine. |
| **Parent study 5** | **Aims:** Determine the clinical, virologic and immunologic outcomes associated with antiretroviral therapy in a longitudinal prospective HIV-2 infected cohort. Assess the role of drug resistance in viruses that emerge in HIV-2 infected subjects with virologic failure. |
|  | **Subjects:** HIV-2 infected adults in Dakar. |

|  | **Funding** | **Study years** | **N** | **Outpatient study clinics** | **Age, median (range)** | **Female (%)** | **HIV positive (%)** |
| --- | --- | --- | --- | --- | --- | --- | --- |
| **Parent study 1** | NIH/NCI  R01 CA62801 | 1994 - 1999 | 1698 | Fann, IHS, Mbour, ASBEF | 32 (18 - 72) | 1126 (66.3) | 852 (50.2) |
| **Parent study 2** | NIH/NIDCR  R01 DE12925 | 1999 - 2004 | 193 | IHS, Mbour, Sebikotane | 37 (21 - 57) | 193 (100.0) | 186 (96.9) |
| **Parent study 3** | NIH/NIAID  R01 AI48470 | 2000 - 2005 | 444 | Fann, IHS | 39 (18 - 63) | 330 (75.2) | 397 (89.8) |
| **Parent study 4** | NIH/NCI  R01 CA111187 | 2005 - 2010 | 472 | Fann, Pikine | 44 (18 - 84) | 472 (100.0) | 173 (37.9) |
| **Parent study 5** | NIH/NIAID  R01 AI 60466 | 2005 - 2015 | 106 | Fann | 49 (21 - 67) | 72 (69.2) | 106 (100.0) |

IHS: Institut d’Hygiene Sociale, Dakar; ASBEF: Association Sénégalaise pour le Bien-Être Familial, Dakar

**Table B in S1: Parent study aims and subjects**

CSW: commercial sex worker
